# Chronic Pain Prevalence and Exposures during Pregnancy

**DOI:** 10.1155/2019/6985164

**Published:** 2019-08-08

**Authors:** Shona L. Ray-Griffith, Bethany Morrison, Zachary N. Stowe

**Affiliations:** ^1^Department of Psychiatry, University of Arkansas for Medical Sciences, Little Rock, AR, USA; ^2^Department of Obstetrics & Gynecology, University of Arkansas for Medical Sciences, Little Rock, AR, USA; ^3^College of Medicine, University of Arkansas for Medical Sciences, Little Rock, AR, USA; ^4^Department of Psychiatry, University of Wisconsin at Madison, Madison, WI, USA

## Abstract

Pregnant women with chronic pain present a unique clinical challenge for both chronic pain and obstetrical providers, and clinical guidelines do not exist. The present study describes the prevalence and management of chronic pain during pregnancy in a perinatal mood disorder clinic. A retrospective chart review of pregnant women who presented to the Women's Mental Health Program at the University of Arkansas for Medical Sciences (UAMS) for an initial evaluation from July 2013 to June 2016 was conducted to obtain demographic and medical information, including pharmacological exposures. Data are described using the mean and standard deviation for continuous data and frequency for categorical data. Pain complaints and medications are presented as counts and percentages. Differences between women with and without chronic pain were assessed by *t*-tests for continuous variables and chi-square analysis for categorical variables. Of the 156 pregnant women, chronic pain conditions were reported by 44 (28.2%). The most common chronic pain complaints included neck and/or back pain (34.1%) and headaches (31.8%). Of subjects with chronic pain, 95.5% were taking at least one prescription medication (mean = 2.6 ± 2.1, range of 0–10). Acetaminophen (43.2%) and opioids (43.2%) were the most common. The complexity of managing maternal benefits of treatment with the risks of fetal exposures presents a uniquely challenging clinical scenario for healthcare providers.

## 1. Introduction

In 2011, the Institute of Medicine released a report on pain estimating that 100 million adults in the United States live with chronic pain conditions [1]. Physical and mental functioning, quality of life, and professional and personal relationships are negatively affected by chronic pain. Moreover, societal stigma around chronic pain and risks associated with the long-term use of pain medications, such as opioids, exacerbates these problems. Women, in comparison with men, experience a greater burden of disease and functional disability as well as lower therapeutic effects with long-term opioid use [2–4]. The perinatal period presents a particularly vulnerable state for women with chronic pain disorders, and data supporting the prevalence, course, and management of perinatal chronic pain disorders are scarce [5]. Chronic pain and obstetrical providers encounter a complex clinical situation where maternal benefits of treatment must be effectively balanced with the risks of maternal illness and fetal exposure to both treatment and maternal illness.

Treatment options for chronic pain disorders during the perinatal period include both pharmacological and nonpharmacological options (e.g., physical therapy); however, uncertainties around treatment impact both the patient and the provider's medical decision-making. For example, among a sample of pregnant women with low back pain, only 32% discussed these symptoms with their prenatal care providers, and 75% of providers did not make any recommendations to manage symptoms [6]. Many women also discontinue pharmacological options immediately upon knowledge of conception due to fears of teratogenic potential.

Another concerning factor includes the limited and discordant reproductive safety data on pharmacological treatments for chronic pain [7]. For example, acetaminophen is a first-line analgesic for pregnant women as it does not produce classic teratogenic effects. However, recent studies have associated perinatal acetaminophen exposure with an increased risk for attention-deficit/hyperkinetic, respiratory, autism spectrum, and testicular disorders in children [8–17]. Opioids are another example as 14–22% of women fill an opioid prescription during pregnancy [18, 19] despite potential adverse neonatal outcomes [20–24]. Moreover, available data support the use of amitriptyline and/or nortriptyline as first-line agents for both nociceptive and neuropathic pain during pregnancy [25–27]; however, use in nonpregnant populations is limited by underutilization by prescribers [28] and patient intolerability [29]. Potential adverse obstetrical and/or neonatal complications are associated with maternal use of NSAIDs and venlafaxine [30], and the use of pregabalin and duloxetine is limited by insufficient reproductive safety data [31]. Notably, antiepileptic exposure in pregnancy is more common for the treatment of pain than epilepsy [32–34]. However, reproductive safety data of antiepileptic drugs are derived from women with epilepsy as well as mood and pain disorders with the majority of studies being confounded by polypharmacy [33–37].

Collectively, reproductive safety data indicate a high potential for adverse outcomes associated with the most commonly used pharmacological treatments for chronic pain, which supports almost exclusive recommendations to use nonpharmacological treatments (e.g., physical therapy and massage). Unfortunately, access to these options may be limited, ineffective, and/or unaffordable. With limited available data to support medical decision-making in women with chronic pain disorders during pregnancy, treatment optimization may depend on reducing concurrent medication exposures, maintaining the minimal effective dose, and decreasing additional exposures associated with risk.

Chronic pain disorders frequently co-occur with mental illness. For example, rates of depression in individuals with chronic pain are twice as high of those without chronic pain [38]—a difference more impactful when comparing women and men [39, 40]. Psychiatrists are often involved in the treatment of individuals with chronic pain given the high rates of comorbid mental illness. Thus, reproductive psychiatrists may find themselves as part of the team managing pregnant patients with chronic low back pain, depression, and fibromyalgia, to name a few. The first step in establishing evidence-based guidelines for the management of chronic pain during the perinatal period is to establish the severity and acuity of the problem. In the present study, we sought to determine the prevalence and assess preevaluation management of chronic pain during pregnancy in a university-based perinatal mood disorder clinic.

## 2. Materials and Methods

A retrospective chart review of all pregnant women who presented to the Women's Mental Health Program (WMHP) at the University of Arkansas for Medical Sciences (UAMS) for an initial evaluation from July 1, 2013, to June 30, 2016, was conducted. The WMHP is an academic mental health program specializing in the treatment of neuropsychiatric illnesses during the perinatal period. The referral base of the WMHP includes psychiatric and obstetrical practices within UAMS and the community throughout Arkansas as well as self-referral. For the current study, subjects were excluded if they were not currently pregnant (i.e., postpartum or preconception), less than 18 years of age, and/or did not consent to having their information used for clinical research investigation at their initial visit. Information was obtained from written and electronic medical records and included demographic information, medical and surgical history (including obstetrical and psychiatric), medication use, and other exposures.

Demographic and obstetrical data are described using the mean and standard deviation for continuous data and frequency for categorical data. Missing data were not included in the total. Differences between groups were assessed by *t*-tests for continuous variables and by chi-square analysis for categorical variables. Chronic pain complaints and current medications are presented as counts and percentages. A *p* value of <0.05 was considered statistically significant. Data were analyzed using Sigma Plot 12.5.

This study was approved by the institutional review board at UAMS.

## 3. Results

A total of 222 pregnant women presented to the WMHP at UAMS for an initial evaluation. Of which, 66 respondents declined participation in research. This resulted in 156 participants for the current analysis. Chronic pain conditions were reported by more than a quarter of respondents (*n* = 44, 28.2%).  Table 1 displays the demographic characteristics of respondents, including by the presence or absence of chronic pain complaints. No differences were found between the groups.

Among respondents with chronic pain, the most common complaints included neck and/or back pain, headaches, and “other” pain complaints (e.g., pelvic and abdominal pain, neuropathic pain, and fibromyalgia). 95.5% (*n* = 42) were currently taking at least one prescription medication, and 59.5% (*n* = 26) were taking two or more medications.

Table 2 lists all medications reported by respondents with chronic pain. The mean number of medications taken was 2.6 ± 2.1 with a range between 0 and 10. More than 20% (*n* = 10) of subjects with chronic pain were concomitantly taking an opioid and benzodiazepine. Nonpharmacological therapy, including physical therapy and transcutaneous electrical nerve stimulation, was reported by 20.5% (*n* = 32).

## 4. Discussion

The literature on chronic pain among pregnant women is limited, and this study aimed at determining the prevalence and preevaluation management of chronic pain during pregnancy in a university-based perinatal mood disorder clinic. A chronic pain condition was self-reported by 28.2% of initial evaluations, and the two most common complaints were neck and/or back pain and headaches. The majority were taking more than one medication at initial presentation with nearly half taking acetaminophen and/or opioids. Nonpharmacological therapy was less common contributing in less than one-quarter of respondents.

With high rates of comorbid pain in people with depression [41, 42], it was not surprising that our study found more than a quarter of patients presenting to a perinatal mood disorder clinic had a comorbid chronic pain condition. Previous studies of chronic prescription opioid use among pregnant women for pain management also identify headaches/migraine and back pain as the most frequent complaints [18, 43]. Although the rate of chronic pain conditions during pregnancy is lower than those in the general population, the authors believe it warrants the need for awareness among chronic pain physicians as well as obstetrical providers. The national opioid epidemic has raised awareness of the complexity of patients with chronic pain disorders, chronic opioid treatment, and opioid use disorders. As a result, providers should be aware of the Center for Disease Control and Prevention (CDC) guidelines for the management of chronic pain disorders [44] as well as the American College of Obstetrics and Gynecology guidelines for the management of opioid use disorder during pregnancy [45].

Our previous work has shown that less than a quarter of reproductive-aged women with long-term opioid prescriptions fill prescriptions for contraceptives [46]. The study population had an unplanned pregnancy rate of 69.5%. Given the rate of unplanned pregnancies in the general United States population is nearly 50% [47], women with chronic pain disorders may have an unmet need for family planning. The CDC recently published guidelines for the prescription of opioids for chronic pain, and recommendations include a discussion of family planning with the initial prescription of opioids [44]. This discussion should include family planning as well as the adverse obstetrical and fetal risks associated with pharmacological and nonpharmacological treatment options. If an unintended pregnancy does occur, a realistic goal is the development of a treatment plan at the earliest possible gestational age to maximize the benefit-to-risk ratio.

Primary fetal development occurs in the first trimester (i.e., up to 14 weeks of gestation), and exposures during that time may pose a risk. In this study, pregnant women presented at about 21 weeks of gestation or during the second trimester—past the first trimester. Moreover, the overwhelming majority of women were taking at least one analgesic medication. Notably, up to 10 pharmacological agents were reported in isolated cases. Application of known reproductive safety data is difficult in patients with chronic pain disorders as the impact of polypharmacy on perinatal outcomes is understudied.

Acetaminophen and opioids were the most commonly used agents—a finding that raises numerous questions. First, are these patients receiving optimal care for chronic pain conditions prior to knowledge of pregnancy? First-line pharmacological options for chronic pain include acetaminophen, nonsteroidal anti-inflammatory drugs, topical agents, tricyclic antidepressants, selective norepinephrine reuptake inhibitors, and gabapentinoids [48–52], while opioids are last-line treatment options. Second, the reproductive safety data of acetaminophen have recently been questioned [8, 9, 11, 15, 16, 53, 54]. Moreover, clinical experience raises concerns of appropriate dosing of acetaminophen as patients often report exceeding recommended dosage instructions and are given sparse guidance from healthcare professionals regarding dosing and presence of acetaminophen in preparations of over-the-counter and prescription medications. Third, the specific risks of opioids for chronic pain syndromes during the perinatal period are unknown. Data concerning obstetrical and neonatal outcomes with opioid exposure in utero are often derived from addiction studies and may not be directly applicable to pain disorder management [55]. Lastly, the epidemic of prescription opioid use in the United States population has extended to pregnant women and is of concern secondary to the adverse effects opioids have on maternal and neonatal health [19, 56, 57]. Comparable to this study, previous studies support an increasing trend of opioid use among pregnant women [19, 56, 57]. Changing physiology in pregnancy may require increased daily opiate dose [58], and prenatal exposure may exceed six months. Both duration of use and dose escalation are associated with increased risk of addiction [59, 60].

Only 20.5% of respondents utilized nonpharmacological treatments. Possible explanations for low utilization of nonpharmacological therapies include a lack of access, efficacy, reproductive safety data, and/or inadequate insurance coverage. Our study did not identify rates of cognitive-behavioral therapy use for chronic pain disorders, but cognitive-behavioral therapy is an effective treatment that should be considered for both chronic pain conditions and many psychiatric disorders in the perinatal period.

The current study has limitations warranting consideration. The population is derived from a specialty clinic and may not be generalizable to populations without psychiatric illnesses. Data were primarily collected from self-reported intake forms and are subject to recall bias. Although self-reported measures have been found to be a strong indicator of pain and a patient's psychosocial profile [61], they are only part of the clinical picture and must be used in conjunction with valid observation-based assessment tools in order to accurately evaluate patients. Finally, this was a retrospective chart review in a clinic focused on neuropsychiatric disorders during the perinatal period, and reports of chronic pain disorders may be underreported as the extent of inquiry by providers may have varied.

## 5. Conclusion

Chronic pain conditions among pregnant women with psychiatric illness are common and likely confer a significant burden of disease to both mother and fetus. The complexity of managing maternal benefits of treatment with the risks of fetal exposures presents a uniquely challenging clinical scenario for both chronic pain and obstetrical providers. Current guidelines for the management of chronic pain emphasize the importance of multimodal approaches, and progress towards effective and safe chronic pain management in pregnancy will require a multidisciplinary, biopsychosocial approach. Future research should examine both independent and dependent effects of pain and its treatment on perinatal outcomes in neuropsychiatric populations.

## Figures and Tables

**Table 1:** Demographic and medical history of pregnant women in a perinatal mood disorder clinic.

Demographics	All (*n* = 156)	Chronic pain complaint		*p*-value
		Yes (*n* = 44)	No (*n* = 112)	
Age (years ± SD)	28.3 ± 5.5	29.1 ± 4.8	27.9 ± 5.8	0.205
Education (years ± SD)	14.3 ± 2.4	14.2 ± 2.1	14.3 ± 2.5	0.606
Race/ethnicity (%)				
White	126 (80.8)	38 (86.4)	88 (78.6)	0.376
Nonwhite	30 (19.2)	6 (13.6)	24 (21.4)	
Marital status (%)				
Married	78 (50.0)	20 (45.5)	58 (51.2)	0.594
Not married	78 (50.0)	24 (54.5)	54 (48.2)	
Medical comorbidity^a^ (%)				
Yes	50 (32.1)	14 (31.8)	36 (32.1)	0.880
No	106 (67.9)	30 (68.2)	76 (67.9)	
Self-reported history of physical, emotional, or sexual abuse (%)				
Yes	94 (61.0)	29 (65.9)	65 (59.1)	0.548
No	60 (39.0)	15 (34.1)	45 (40.9)	
Tobacco use (%)				
None	69 (44.2)	21 (47.7)	48 (42.9)	0.520
Current use	42 (26.9)	9 (20.5)	33 (29.5)	
History of use	45 (28.8)	14 (31.8)	31 (27.7)	
Alcohol use (%)				
None	62 (39.7)	19 (43.2)	43 (38.4)	0.652
Current use	2 (1.3)	1 (2.2)	1 (0.9)	
History of use	92 (59.0)	24 (54.5)	68 (60.7)	
Self-reported illicit drug use (%)				
None	81 (51.9)	25 (56.8)	56 (50.0)	0.383
Current use	15 (9.6)	2 (4.5)	13 (11.6)	
History of use	60 (38.5)	17 (38.6)	43 (38.4)	
Obstetrical information				
Severity (number ± SD)	2.8 ± 1.8	3.1 ± 1.9	2.7 ± 1.7	0.139
Parity (number ± SD)	1.1 ± 1.2	1.4 ± 1.3	1.0 ± 1.1	0.083
Gestational age (weeks ± SD)	21.2 ± 8.8	21.2 ± 9.5	20.3 ± 8.5	0.620
Trimester at initial evaluation (%)				
1^st^	48 (31.0)	14 (31.8)	34 (30.6)	0.138
2^nd^	66 (42.6)	14 (31.8)	52 (46.8)	
3^rd^	41 (26.5)	16 (36.4)	25 (22.5)	
Planned pregnancy (%)				
Yes	46 (30.5)	11 (25.6)	35 (32.4)	0.531
No	105 (69.5)	32 (74.4)	73 (67.6)	
Desired pregnancy (%)				
Yes	76 (50.3)	22 (51.2)	54 (50.0)	0.870
No	15 (9.9)	5 (11.6)	10 (9.3)	
Mixed feelings	60 (39.7)	16 (37.2)	44 (40.7)	

^a^Medical comorbidities include hypertension, diabetes, asthma, and thyroid conditions.

**Table 2:** Medication exposures among pregnant women with chronic pain disorders.

Medication class	*N* (%)	Individual medications	*N* (%)
Opioids	19 (43.2)	Buprenorphine	1 (2.3)
		Fentanyl	1 (2.3)
		Hydrocodone	13 (29.5)
		Oxycodone	6 (13.6)
		Tramadol	4 (9.1)
Acetaminophen	19 (43.2)	Acetaminophen	19 (43.2)
Sedative/hypnotic	16 (36.4)	Alprazolam	6 (13.6)
		Clonazepam	3 (6.8)
		Diazepam	5 (11.4)
		Lorazepam	1 (2.3)
		Zolpideim	6 (13.6)
Antiepileptic drugs	13 (29.5)	Gabapentin	6 (13.6)
		Lamotrigine	4 (9.1)
		Pregabalin	1 (2.3)
		Topiramate	2 (4.5)
Muscle relaxant	7 (15.9)	Carisoprodol	3 (6.8)
		Cyclobenzaprine	3 (6.8)
		Methocarbamol	1 (2.3)
Steroid	7 (15.9)	Steroid	7 (15.9)
Nonsteroidal anti-inflammatory drugs	3 (6.8)	Etodolac	1 (2.3)
		Ibuprofen	1 (2.3)
		Naproxen	1 (2.3)
Selective serotonin reuptake inhibitors	11 (25.0)	Citalopram	2 (4.5)
		Escitalopram	2 (4.5)
		Fluoxetine	5 (11.4)
		Sertraline	2 (4.5)
Selective norepinephrine reuptake inhibitors	5 (11.4)	Desvenlafaxine	1 (2.3)
		Venlafaxine	4 (9.1)
Tricyclic antidepressants	1 (2.3)	Doxepin	1 (2.3)
Norepinephrine reuptake inhibitor	1 (2.3)	Bupropion	1 (2.3)

## Data Availability

The clinical data used to support the findings of this study have not been made available due to patient privacy.
